# Sir John Tomes

**Published:** 1895-12

**Authors:** 


### SIR JOHN TOMES.

Died, July 29, 1895, at his home, Upwood Gorse, Caterham Valley, Surrey, England, Sir JOHN TOMES, F. R. S., F. R. C. S., L. D. S. Eng., in the eighty-first year of his age.

John Tomes was born on March 21, 1815, at Weston-on-Avon, Gloucestershire. He commenced the study of medicine under the tutelage of a practitioner at Evesham, to whom he was apprenticed in 1831. He entered the Medical schools of King's College and Middlesex Hospitals. He was house surgeon at the latter for two years, and passed the first examination for the degree of M. B. at the University of London in 1839. He was dental surgeon to King's College Hospital for some years, and resigned that post for the same one at Middlesex Hospital. During his house surgeonship at Middlesex he invented forceps adapted to the necks of the different teeth. which caused Sir Thomas Watson to advise him to adopt dental surgery as his profession. In 1840 he commenced practice at 41 Mortimer St. (now Cavendish Place), London, and for some years, even after his marriage in 1844, he had a hard struggle to make both ends meet. In 1845 he invented a machine for copying in ivory irregular curved surfaces, which obtained the gold medal of the Society of Arts. In 1845 he also delivered the course of lectures at Middlesex which marked a new era in dentistry, and when published in the *Medical Gazette*, and afterward, in 1848, in book form, at once made his reputation as a scientific observer. In relation to these lectures there is this note in his diary: "I am resolved never to deliver any more lectures unless I have a class of six."

Following the announcement of the discovery of the anæsthetic use of ether, in 1846, by Dr. Morton, of Boston, Mr. Tomes at once took it up, and successfully used it in his work at the hospital. In 1843 he joined a few leading dentists in memorializing the College of surgeons to recognize the claims of dentistry to be considered a department of general surgery The attempt at that time failed, from lack of support upon the part of the medical profession and from the absence of popular sentiment in its favor. The interest of Mr. Tomes was unabated, however, and he continued his labors and interest in the cause he had championed until his object was accomplished, in 1859, by the establishment of a department of dental Surgery at the College of Surgeons.

On June 6, 1850, he received the Fellowship of the Royal Society, as being "distinguished for his acquaintance with the sciences of anatomy and physiology."

In 1859 he published his "System of Dental Surgery," a standard work so well and favorably known as to require no further comment herer In 1862 a strong feeling which had arisen that the services of Mr. Tomes merited some public token of the gratitude of the profession took definite form in the presentation to him, on July 16, of that year, of a handsome service of plate by several of his brother practitioners, in acknowledgement of the many valuable services he had rendered to his profession.

As he had been actively interested in securing the recognition of dental surgery as a department of general surgery, by the establishment of a more thorough educational curriculum for dental students, so in like manner his interest and activities were enlisted in the movement toward making the new standard of education obligatory by appropriate legislation. With untiring zeal he labored with others to that end, and on July 22, 1878, the Dentist Act became law. Sir John Tomes was the first to inscribe his name upon the Dental Register.

On July 26, 1880, the profession presented Mr. Tomes with a portrait of himself, by Macartney. In 1883, the very high compliment of Honorary Fellowship in the college of Surgeons was conferred upon Mr. Tomes. The college having the right to confer its fellowship upon but two persons annually; Mr. Tomes and Professor Huxley were selected for the honor as representative men of science, worthy of special regard. In May of 1886 he received and accepted the offer of knighthood "for eminent services rendered his profession."

In 1893 it became known that the golden wedding anniversary of Sir John and Lady Tomes occurred upon February 15, of that year. The opportunity thus afforded to the profession of once more showing its appreciation and regard was enthusiastically welcomed. A deputation went to Upwood Gorse, and Mr. Brunton presented to Lady Tomes a silver-gilt inkstand, on behalf of the wives of the members of the profession. Sir Edwin Saunders announced the foundation of a triennial "Sir John Tomes" prize, to be awarded by the College of Surgeons, in recognition of satisfactory scientific work by Licentiates in Dental Surgery, and Mr. Hutchinson read an appropriate address on illuminated vellum, signed by a large number of dentists.

In the summer of 1894 his health, which for years had not been robust, was impaired still further by what appeared to be indigestion. This increased greatly, and in May his illness assumed a more serious form, and gradually growing worse, in spite of the best medical care, he died July 29, and was buried at St. Mary's, Upper Caterham.

Sir John Tomes, during the course of his career, filled the highest positions within the gift of his colleagues, and in each of these he evinced qualities of leadership and capability which raised him to the highest place in their esteem. His love of fairness and right, and the judicial qualities of mind which were among his strongest attributes, were qualities which gained for him the respect and confidence even of those to whom he was at times opposed.

His numerous contributions to the science and art of dentistry stamp him as a scientific observer of the highest order of merit, and the sum total of his characteristics, as recorded in his valuable life-work, will ever maintain his splendid example as an ideal for future generations of his followers to emulate.

We are indebted to the *Journal of the British Dental Association* for the greater part of the data upon which the foregoing sketch is based.—*Dental Cosmos.*

